# A Prospective Controlled Study on Long-Term Outcomes of Facial Lacerations in Children

**DOI:** 10.3389/fped.2020.616151

**Published:** 2021-02-12

**Authors:** Sonja Fontana, Clemens M. Schiestl, Markus A. Landolt, Georg Staubli, Sara von Salis, Kathrin Neuhaus, Christoph Mohr, Julia Elrod

**Affiliations:** ^1^Department of Pediatric Emergency Medicine, University Children's Hospital Zurich, Zurich, Switzerland; ^2^Children's Research Center (CRC), University Children's Hospital Zurich, Zurich, Switzerland; ^3^Division of Plastic and Reconstructive Surgery, University Children's Hospital Zurich, Zurich, Switzerland; ^4^Department of Psychosomatics and Psychiatry, University Children's Hospital Zurich, Zurich, Switzerland

**Keywords:** suture, glue, pediatrics, scar, blinded, outcome, POSAS, VSS

## Abstract

**Background:** Although skin adhesives have been used for decades to treat skin lacerations, uncertainty remains about long-term results, and complications.

**Methods:** In this prospective, controlled, single-blinded, observational cohort study, outcomes were assessed by five plastic surgeons with standardized photographs at 6–12 months using a modified Patient and Observer Scar Assessment Scale (POSAS) and Vancouver Scar Scale (VSS); additionally, the POSAS was performed by the patients/caregivers and the physician; pain, requirement of anesthesia, treatment time, costs, complications, and quality of live (QoL) were assessed.

**Results:** A total of 367 patients were enrolled; 230 were included in the main analysis; 96 wounds were closed using tissue adhesives (group 1); 134 were sutured (group 2). Assessment by the independent observers revealed an improved mean modified overall POSAS score in group 1 in comparison with group 2 [2.1, 95% CI [1.97–2.25] vs. 2.5, 95% CI [2.39–2.63]; *p* < 0.001, d = 0.58] and mean VSS score [1.2, 95% CI [0.981–1.34] vs. 1.6, 95% CI [1.49–1.79], *p* < 0.001, d = 0.53]. At the early follow-up, dehiscence rate was 12.5% in group 1 and 3.7% in group 2 (*p* < 0.001); later on, one dehiscence remained per group. Mild impairment of QoL was found at the early follow-up in both groups, with no impairment remaining later on. Duration of treatment and treatment costs were lower in group 1.

**Conclusion:** Both modalities of wound closure yield favorable esthetic results, and complications are rare. Adhesives are more cost-effective, and its application is less time-consuming; therefore, tissue adhesives offer considerable advantages when used appropriately.

**Trial Registration:** Public trial registration was performed at www.ClinicalTrials.gov (Identifier: NCT03080467).

## Introduction

The ideal therapy for pediatric injuries in the emergency room should fulfill the following criteria: it should be fast, non-traumatizing with no or little pain, it should be safe and lead to good long-term results. Lacerations are one of the most common reasons for pediatric patients to seek medical care (1). These wounds need to be cleaned, and the wound edges should then be adapted to ensure optimal healing and prevent infection or hypertrophic scarring (2). Traditionally, sutures have been used to re-approximate the wound edges. However, due to their development status, toddlers are often non-compliant and do not tolerate pain associated with wound adaptation or suturing (2, 3). Therefore, this procedure often requires general anesthesia or sedation, which in turn is accompanied by standard anesthetic risks and is time-consuming (4). Furthermore, according to the Centers for Disease Control 50% of the emergency departments (EDs) are often overcrowded, and waiting time is often perceived to be too long by caregivers and patients (5).

On the basis of these difficulties, tissue adhesives have become more and more popular as an alternative method for wound closure, but at the same time, they are frequently criticized as yielding inferior results or their use is limited to certain types of injuries, such as superficial lacerations with low wound tension, with good wound approximation of short length and excluding those located in high-mobility sites (6–8). To date, only limited controlled studies are available on whether this technique is actually less traumatizing, safe, cost-effective, and leads to good long-term results (9).

A Cochrane review (9) analyzed suture vs. glue for laceration repair in children and found tissue adhesive to be an acceptable alternative to suture. However, none of the 13 studies included reported data from larger cohorts on long-term outcomes, the largest study with a follow-up period of 1 year included 84 patients. Results from the meta-analysis showed significant random-effect differences calling for further clinical studies. Furthermore, none of the studies performed a blinded analysis by external, independent surgeons or included patient perspectives and cost-effectiveness.

The primary goal of this systematic and blinded observational cohort study is to test our hypothesis that the long-term outcome of facial laceration repair using tissue adhesives is not inferior to the use of sutures in pediatric patients. Additionally, complications, quality of life (QoL), cost-effectiveness, pain, and necessity of anesthesia are to be assessed.

## Patients and Methods

A prospective, controlled, single-blinded observational cohort study was designed to compare the outcomes of the use of tissue adhesive and suture for the repair of lacerations on the head in children. Ethical approval was obtained from the local ethics committee (BASEC-No 2016-01304). Enrollment took place at the University Children's Hospital in Zurich, Switzerland, between July 2017 and August 2018.

Pediatric patients (age 0–16 years) who presented to the ED with a facial laceration were eligible. Among those patients with lacerations on the lip and tongue, polytrauma patients and those with underlying or concomitant conditions that might interfere with wound healing (e.g., history of keloid formation, current oral steroid therapy, and diabetes) were excluded. Informed written consent from the legally authorized representatives of patients < 14 years or from the patient ≥ 14 years of age was obtained by the research study team. The study comprised three visits and a blinded online evaluation by five independent surgeons.

### Study Visits

#### Primary Treatment (Visit 1)

Laceration repair was performed in a standardized manner. Whenever necessary, local anesthesia was provided using either topical LET (lidocaine, epinephrine, tetracaine) gel or infiltration with lidocaine 1%. If required, general anesthesia or sedation using nitrous oxide, midazolam, and/or ketamine was provided. Wound closure was performed using either non-absorbable sutures (Ethilon®) or absorbable sutures (Vicryl®) or ethyl-2-cyanoacrylate tissue adhesive (Epiglu®). It was at the discretion of the treating physician to apply either sutures or tissue adhesives, considering patient age, compliance, the patient's/caregiver's preferences, and wound characteristics, such as difficulties in adapting the margins or perioral injuries, in which both were preferably sutured. Pain was recorded from the patient or—if under 4 years of age—as proxy report by the caregiver using Visual Analog Scale (VAS). Patients and care-givers were asked to keep the wound dry and clean for one week.

#### Early Follow-Up (Visit 2)

Patients were asked to return to the ED after 5–10 days. Whenever applicable, suture material was removed. Complications were assessed by means of the Wound Evaluation Score (WES). Skin-related QoL was quantified using the Children's Dermatology Life Quality Index (CDLQI). Standard scar care instructions were given to the patients and caregivers, comprising daily scar tissue massage using a skin-sensitive cream and application of sunscreen lotion with a high protection factor before each exposure to the sun for the course of one year.

#### Late Follow-Up (Visit 3)

Late follow-up was scheduled 6–12 months following laceration repair. The outcome was assessed by means of the CDLQI and the Patient and Observer Scar Assessment Scale (POSAS).

#### Independent Scar Evaluation

Outcomes after 6–12 months were assessed by five independent, blinded plastic surgeons. At the time of evaluation, none of them were employed at the study center or otherwise involved in the study. A custom, browser-based evaluation tool was created to assess all photographs. The picture sequence was randomized and displayed in a consistent order. The specialists were asked to rate each picture using a modified POSAS Observer scale and a modified VSS. As the items pliability (POSAS) and stiffness (VSS) cannot be assessed on a picture, those have been deleted from the original scales, thus resulting in the “modified VSS” and the “modified POSAS” scales. Provided answers were automatically exported to a database for data analysis.

### Outcome Measures

#### Visual Analog Scale

The VAS is a validated tool for quantitative detection of pain, developed for patients from the age of 4 years. The VAS consists of a continuous line, reaching from score 0 “no pain at all” to score 10 “worst possible pain” (10, 11).

#### Patient and Observer Scar Assessment Scale

The POSAS is a validated scar assessment tool, comprising an observer and a patient scale (12–15). Each of them contains six parameters plus an overall rating. Scales range from 1 (normal skin/no complaints) to 10 (worst scar imaginable/worst symptoms). In preschoolers, the questionnaire was answered by the caregiver.

#### Vancouver Scar Scale

The VSS is a validated tool for scar assessment, which merely records the observer's rating of the scar (16). It contains four parameters, leading to a total score ranging from 0 to 13, where 0 is the best possible value and 13 the worst possible score (17, 18).

#### Wound Evaluation Score

The WES is a rating scale addressing six clinical categories; a score of 6 is considered optimal, whereas a score of ≤ 5 is suboptimal (19).

#### Assessment of Complications

Complications were assessed by the study personnel (medical staff) at early and late follow-ups using the four binary categories: no complications, wound dehiscence, wound infection, and others. The use of procedural sedation was not considered an adverse event.

#### Children's Dermatology Life Quality Index

The questionnaire measures the impact of a skin disease on QoL (20, 21). A cartoon version is available for children > 4 years (22); below this age, a proxy version is available. It contains 10 questions, leading to a maximum sum score of 30. Higher scores indicate greater QoL impairment. As suggested by Waters et al. (23), the following severity bands were used: 0–1, “no effect”; 2–6, “small effect”; 7–12, “moderate effect”; 13–18, “very large effect”; and 19–30, “extremely large effect” on QoL.

#### Photographic Documentation

Standardized photographic documentation of the laceration, respectively, the scar, was carried out at each visit at a set distance of 30 cm using a Nikon D810 photo camera (37 megapixels) with a Nikon AF-S Micro NIKKOR 60 mm 1:2.8 G ED lens, macro setting with no flash, with a Kaiser KR 90 LED ring light, designed to ensure the same brightness conditions for all photos. The size of the wound was recorded with a standard scale. All photographs were edited by a medical photographer using Adobe Camera Raw for Photoshop CC (Version 9.12.1). Exposure and brightness were adjusted, white balance was performed, and contrast was optimized. Subsequently, pictures were cropped consistently using Photoshop CC (2019, Version 20.0.8), and tone curves were adjusted automatically on the basis of gradation curves. In a few cases, the filter “sharpen–unsharp mask” was applied.

#### Time and Cost-Effectiveness

Time from wound disinfection to completed laceration repair was documented. In contrast, costs were not assessed individually for each case. Instead, the case-based lump sums for each of the two treatment options (tissue adhesive vs. suture) according to the current tariff of the Swiss health system (www.tarmed-browser.ch) were used for cost calculations.

### Data Analysis

Incomplete data sets were excluded from the analysis. Patients lost to early follow up were replaced, whereas patients lost to late follow up were not replaced. A power analysis was performed based on preliminary POSAS results, using a two-tailed *T-*test and assuming an effect size of 0.5, leading to a sample size of 105 patients for each group (sutured vs. glue), with a power of 95% and 5% significance.

Data were analyzed using SPSS statistical software for Macintosh (Version 25), after removing sensitive data and anonymizing all patients. Demographics, location, and characteristics of skin lacerations are presented descriptively; differences in outcome measures [VAS, (modified) POSAS, (modified) VSS, CDLQI, WES, and complications] by treatment group were examined by means of unpaired *t-*tests and chi-square tests. Interrater reliability was expressed by means of the intraclass correlation coefficient (ICC). ICC (2.1) was applied for single raters and ICC (2, k) for average raters. For all analyses, statistical significance was defined as p ≤ 0.05, and 95% confidence intervals and Cohen's d were indicated whenever applicable. The figures were plotted using Python (Matplotlib 3.1.1, 2019) (24).

## Results

### General Characteristics

During the study period of 392 days, 1,447 patients presented to our ED with facial lacerations, of which 320 were initially enrolled. Finaly, 286 (20%) participated in the study. Of those, 56 participants were excluded from the main analysis due to a lack of follow-up or missing data, leading to a final number of 230 patients, 101 (44%) female and 129 (56%) male. Mean age was 4.0 ± 2.7 years. Fourteen percent were Fitzpatrick type 1, 39% type II, 30% type III, 1% type IV, 5% type V, and 2% type VI. A total of 224 (97%) wounds appeared clean, and six (3%) were macroscopically contaminated.

A total of 96 (42%) were closed using tissue adhesive (group 1), and 134 (58%) were sutured (group 2). The two treatment groups did not differ significantly in terms of gender (*p* = 0.42), child age (*p* = 0.054), skin type (*p* = 0.50), and wound contamination (*p* = 1).

### Scar Outcomes

**Figure 1:**
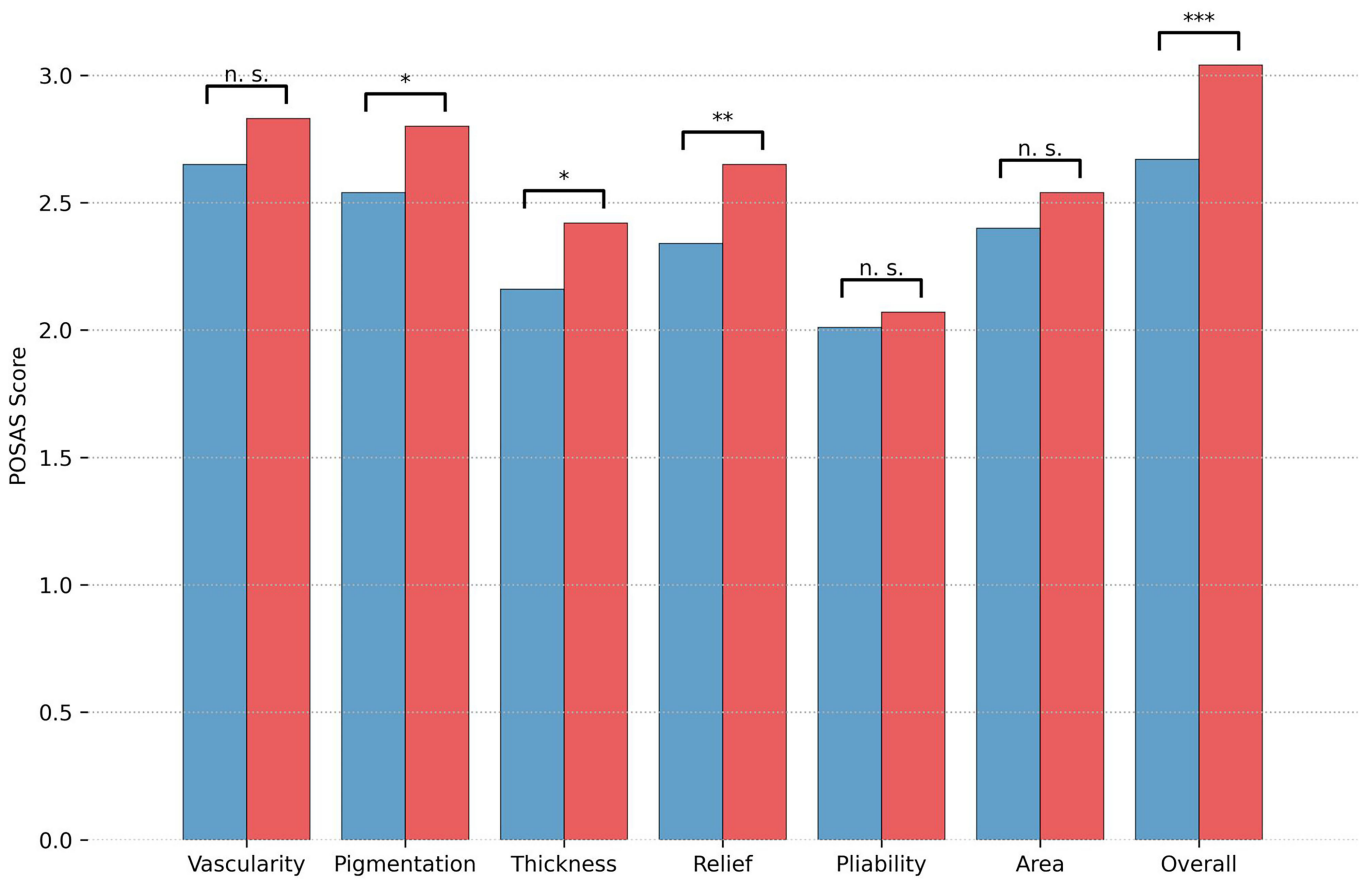
Mean Observer score of the Patient and Observer Scar Assessment Scale (POSAS) at late follow-up (at 6–12 months). Results are depicted separately by treatment group (blue = group 1, tissue adhesive; red = group 2, suture) for each of the POSAS items and for the POSAS Overall score; and significance levels are indicated. Significance levels are indicated (*p < 0.05, **p < 0.01, ***p < 0.001).

Mean time of the late follow-up at 6–12 months was 253.0 ± 52.2 days in group 1 and 255.7 ± 47.1 days in group 2 (*p* = 0.67). The mean overall POSAS Observer score at the late visit was lower (improved) in group 1 compared with group 2 [mean overall score group 1, 2.7, 95% CI [2.54–2.8]; group 2, 3.0, 95% CI [2.91–3.17]; *p* < 0.001, d = 0.52]. The single items pigmentation, thickness, and relief were also significantly lower in group 1 (see Figure 1). Patient overall score at 6–12 months did not differ significantly between the two groups [group 1 = 3.4, 95% CI [3.02–3.8], group 2 = 3.5, [3.22–3.84]; *p* = 0.6, d = 0.06], nor did any of the single items.

### Online Evaluation by Physicians

**Figure 2:**
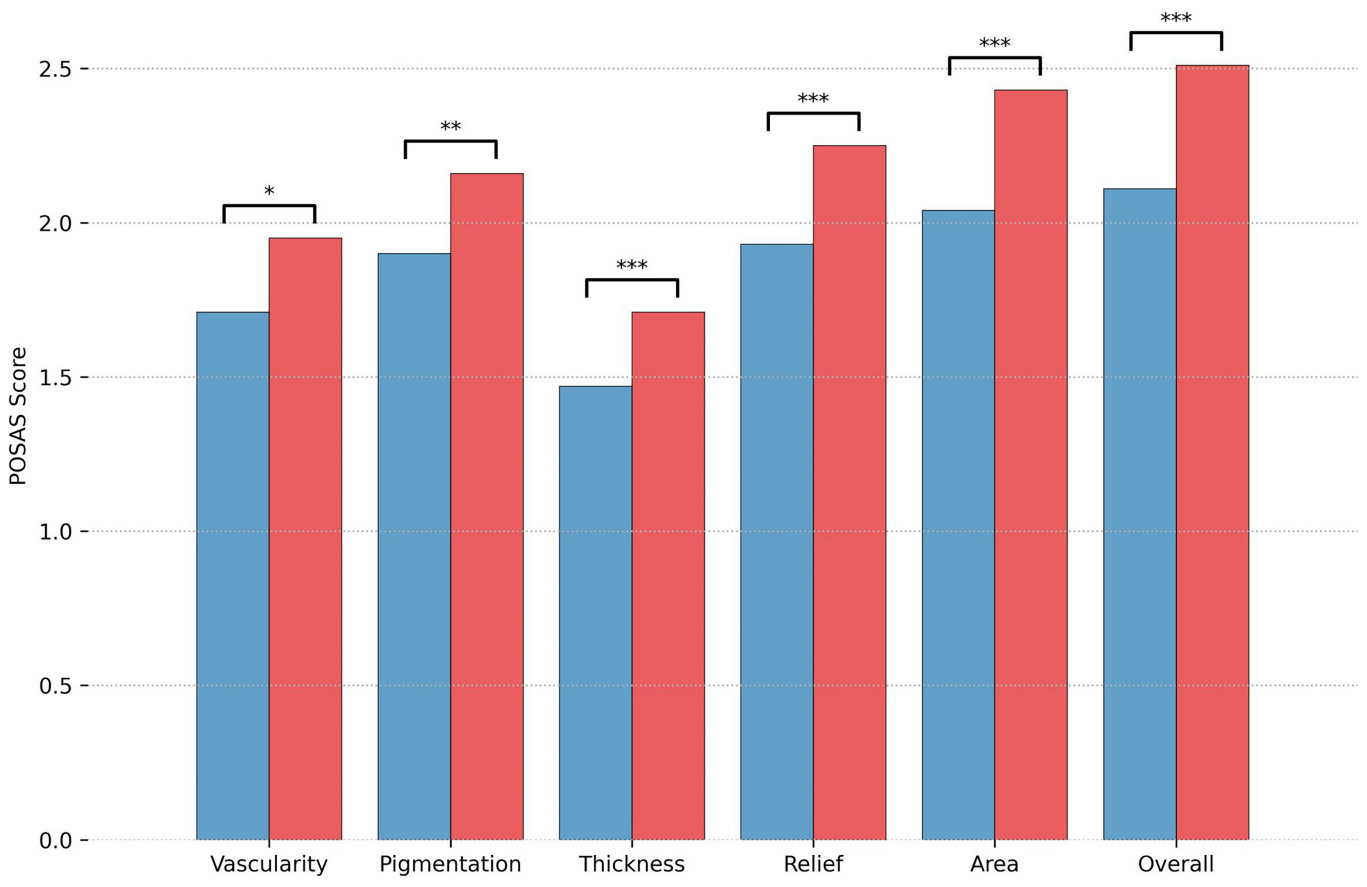
Results of the online evaluation of 230 scars by independent surgeons for mean modified Observer score of the Patient and Observer Scar Assessment Scale (POSAS) are illustrated separately by treatment group (blue = group 1, tissue adhesive, red = group 2, suture). Significance levels are indicated. Significance levels are indicated (*p < 0.05, **p < 0.01, ***p < 0.001).

**Figure 3:**
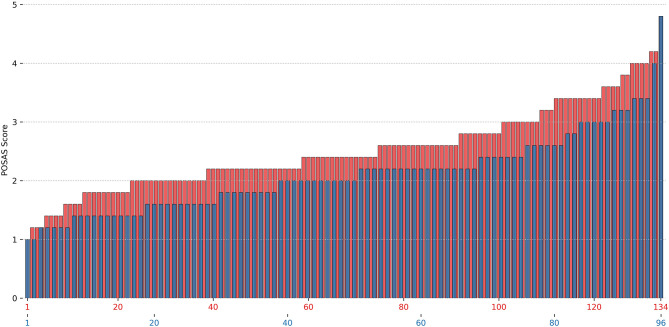
Mean modified total score of the Patient and Observer Scar Assessment Scale (POSAS) shown separately for each of the 230 scars by online evaluation. The mean modified POSAS Total score as evaluated by the five independent, blinded specialists in plastic surgery is shown separately for each of the 230 scars. Results are illustrated by treatment group (blue = group 1, tissue adhesive, red = group 2, suture).

**Figure 4:**
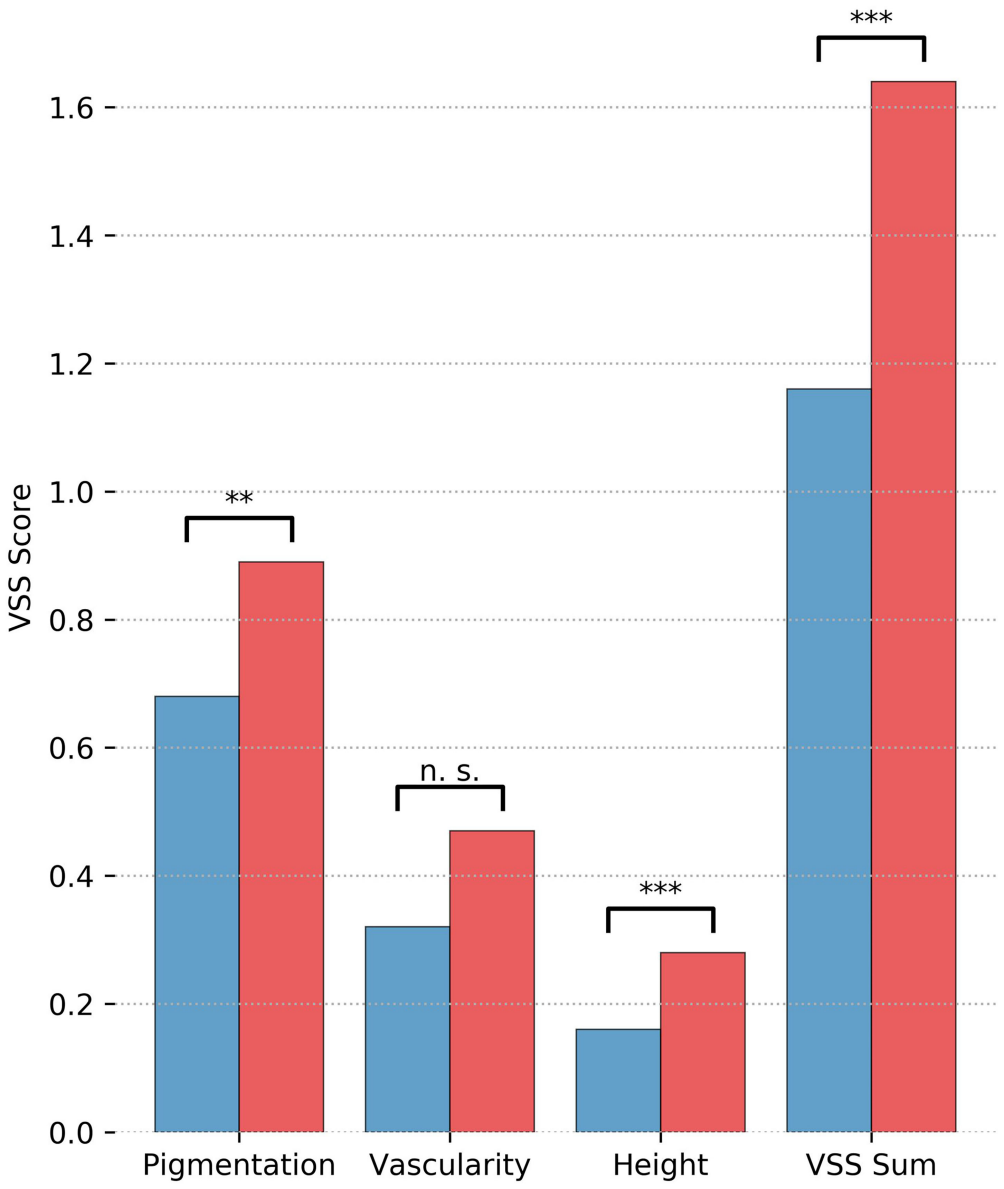
Results of the online evaluation of 230 scars by independent surgeons for mean modified Vancouver Scar Scale Scores are illustrated separately by treatment group (blue = group 1, tissue adhesive, red = group 2, suture). Significance levels are indicated. Significance levels are indicated (**p < 0.01, ***p < 0.001).

**Figure 5:**
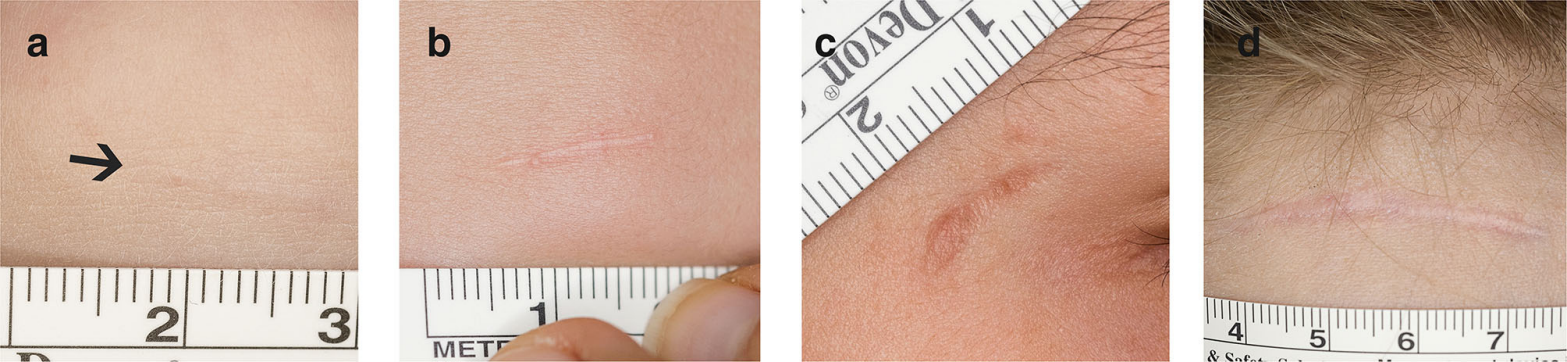
Exemplary range of photographs scored with the POSAS. This is a compilation of four photographs of facial lacerations, scored by one of the online observers using the POSAS and merely serves for a better illustration [POSAS Observer scale, overall opinion = **(a)** 1, **(b)** 3, **(c)** 5, and **(d)** 7].

Online evaluation of the scars by five independent and blinded surgeon specialists revealed a lower mean modified overall POSAS Observer score over all five observers in group 1 (2.1, 95% CI [1.97–2.25]) as compared with group 2 [2.5, 95% CI [2.39–2.63]; *p* < 0.001, d = 0.58]. In addition, all single items scored significantly lower in group 1 (see Figure 2). The distribution over all scars is shown in Figure 3. Mean modified overall VSS score by online evaluation was significantly lower (improved) in group 1 in comparison with group 2 [1.2, 95% CI [0.981–1.34] vs. 1.6, 95% CI [1.49–1.79]; *p* < 0.001, d = 0.53]. The single items pigmentation and height also differed significantly between the two groups (see Figure 4). For illustration purposes, Figure 5 shows an exemplary range of the POSAS Observer scale overall opinion in ascending order.

Interrater reliability for POSAS was ICC (2.1) = 0.33 for single raters, ICC (2, k) = 0.71 for average raters, and ICC(2.1) = 0.45 and ICC(2, k) = 0.81, respectively, for single and average raters for VSS.

### Painfulness of Treatment and Analgesia/Anesthesia

During wound closure, the mean VAS scoring in group 1 was 1.7, 95% CI [1.29–2.09], and in group 2 was 1.7, 95% CI [1.35–2.03]; *p* = 0.99; d = 0.00. In most children, local anesthesia using LET gel was sufficient or no analgesia was necessary at all. Three children in group 1 and 53 children in group 2 required procedural sedation (*p* < 0.001) (20 × nitrous oxide 50%, 36 × midazolam).

### Wound Evaluation Scale

The wound evaluation at early follow-up revealed a good WES for both groups [group 1, 5.40, 95% CI [4.97–5.84]; group 2, 5.84, 95% CI [5.66–6.02]; *p* = 0.07; d = 0.27].

### Complications at Early and Late Follow-Up

At the early follow-up visit, one (1.0%) patient in group 1 had a wound infection, and 12 (12.5%) children presented with a wound dehiscence. In group 2, wound infections occurred in three (2%) cases and dehiscence in five (4%) patients. The differences in rates of dehiscence were significant (*p* = 0.01); the differences in infections were not (*p* = 0.50). Two of the three wound infections were treated with topical antimicrobial ointment only and resolved without sequelae, whereas the third case—a wound that had been sutured—required surgical wound revision under general anesthesia, a course of oral antibiotics, and several visits to our outpatient department.

Concerning wound dehiscence, conservative treatment using wound closure tapes was sufficient in most cases. Only two children in group 1 required secondary suturing, and one child from group 2 required surgical wound revision.

Besides wound infections and dehiscence, one other complication arose. A 2-year-old girl was injured by the surgeon with his scalpel on a digit due to an uncontrolled movement of the child during removal of the sutures. This injury required wound care and an additional follow-up appointment. The overall rate of complications at the early follow-up was 14% (13/96) in group 1 and 7% (9/134) in group 2 (*p* = 0.08).

At the late follow-up, one wound dehiscence was seen in each of the two groups. Further, a 2-year-old patient in group 1 developed pruritus in the affected area in the absence of apparent signs of allergy or eczema. Thus, the overall rate of complications at the late follow-up was 2% (2/96) in group 1 and 1% (1/134) in group 2 (*p* = 0.38).

### Quality of Life

Mean CDLQI at the early follow-up was 2.8, 95% CI [2.24–3.3] in group 1 and 2.9, 95% CI [2.39–3.37] in group 2 (severity band: small effect); *p* = 0.75, d = 0.04. At the late follow-up, mean CDLQI was 0.3, 95% CI [0.046–0.534] in group 1 and 0.6, 95% CI [0.132–1.01] in group 2 (severity band: no effect); *p* = 0.08, d = 0.4.

### Assessment of Time and Cost-Effectiveness

Mean duration of treatment was 6.3 min, 95% CI [5.49–7.11] in group 1 and 11.6 min, 95% CI [10.4–12.8] in group 2; *p* < 0.001, d = 0.91. According to the current tariff of the Swiss health system, the overall cost of a pediatric emergency visit for the repair of a skin laceration was 236 CHF (US$244, conversion rate applicable on 2020-04-18) for tissue adhesive and 355 CHF (US$367) for suture.

## Discussion

The aim of this study was a systematic comparison of short-term and long-term complications and esthetic outcomes of facial lacerations in children, treated with either tissue adhesive or suture. Our analysis found good esthetic outcomes in both groups with low scar rating scores. Yet statistical analysis of the treatment modalities revealed a slightly, improved overall (modified) POSAS and VSS scores, for tissue adhesives in comparison with sutures in the long-term. There was no significant difference in the overall rate of complications in the groups, at neither the early nor late follow-up. Yet cost- and time-effectiveness were in favor of tissue adhesives.

### Complications

In our cohort, the rate of short-term and long-term complications was low in both groups, with the differences between the groups being insignificant. In particular, the wound infection rate was 1% for tissue adhesive and 2% for suture; of all those cases only one involved major sequela (surgical revision). In a meta-analysis, overall infection rate after wound closure with tissue adhesive was reported as 1.1% compared with 0.7% for other wound closure techniques (25).

At 5–10 days post-treatment, the rate of wound dehiscences was almost four times higher in the tissue adhesive group than in the suture group. Of note, almost all resolved without requiring significant treatment and without sequelae, and the rate of wound dehiscences was equal for both groups for the late follow-up visit. This finding suggests early wound dehiscence to be of little importance with respect to long-term outcome. In contrast to our study, a Cochrane review (9) summarizing the available evidence of the outcome of tissue adhesives on traumatic lesions—in different anatomical regions of the body—in a total of 13 studies (2 including only adults and 11 studies also included children) observed a small but statistically significantly increased rate of dehiscence in wounds treated with tissue adhesives. As opposed to our study, most of the included studies had follow-up times of 3 months only or had significantly smaller sample sizes. A period of 3 months only seems to be too short because, according to our results, wounds repaired with tissue adhesive tended to result in a good long-term outcome, despite having an initially greater width. A recent retrospective review that included 1,804 children with repair of a facial/head laceration with tissue adhesive in the 30 days following repair reported higher dehiscence rates for chin lacerations compared with other facial localizations, the risk however not being statistically different between the two treatment modalities (7). Importantly however, lacerations in high mobility sites, laceration of more than 5 cm length, and deep lacerations extending to the muscle were excluded from the use of tissue adhesives in this cohort. Other studies reported similar total rates of complications, in particular infection and dehiscence after 2 months in low-tension, superficial and short lacerations (<2.5 cm) of the face in minors (26), respectively, 3–12 months in patients <14 years of age with simple lacerations, excluding for example, large wounds or areas of high skin tension (6).

### Esthetic Short- and Long-Term Outcomes

In our opinion, objective results using the VSS and POSAS can only be obtained if blinded, non-biased scar assessment is performed by multiple observers. Therefore, photographic evaluation was performed by five plastic surgeons. The hereby achieved ICCs propose that scar evaluation based on an online photographic scar assessment tool is feasible. Several other authors used photographic evaluation of linear scars in the past (2, 27, 28). Quinn et al. (2) reported on a good intraobserver and interobserver agreement (VAS: ICC = 0.94 and ICC = 0.75) for surgeons rating pediatric facial lacerations, and a later publication revealed a good intraobserver agreement (ICC = 0.93) and showed an excellent agreement between research nurses (κ = 0.79, 95% CI [2.39–2.63]) (29). Moreover, Kantor revealed scar scale ratings using photographs to be largely equivalent to live patient assessment in post-operative linear scars (27). In contrast, in her study assessing burn scars from photographs using the POSAS, Manchester Scar Scale, and modified VSS, Simons et al. (30) achieved ICCs of 0.71–0.87 (in-person) and 0.72–0.77 (using photographs) for multiple raters, however, with a low agreement between photographic and in-person assessment.

### Procedural Pain

Surprisingly, the use of tissue adhesive and suture resulted in similar pain scores. However, the informative value is limited because group 1 had significantly less anesthesia and procedural sedation. In most cases, tissue adhesive was applied without any analgesia or with LET gel alone. This is consistent with other studies (2, 31–33). According to Barnett (34), doctors, nurses, and parents reported tissue adhesive as being less painful, while pediatric patients rated pain equally for tissue adhesives and suturing. Indeed, manipulation to re-adapt the wound margins before applying tissue adhesive can be painful, and tissue adhesive may lead to a burning sensation due to polymerization (26, 35–37). In our case, the use of nitrous oxide or midazolam did not lead to any adverse events, yet requiring significantly less procedural sedation is a major advantage of tissue adhesives, given the potential adverse events. In a study in 2015, using nitrous oxide for procedural sedation in 101 children resulted in 10 adverse events (9.9% of all cases), of which most were vomiting (3.9%) and experienced nausea (2.9%) and light-headedness, hyperventilation, and abdominal pain (each 0.9%) (38).

### Quality of Life

Prior studies (20, 39) reported common skin diseases like atopic eczema or molluscum contagiosum to have a large effect on QoL in a significant proportion of children. In a study conducted in 82 adults with scars, Brown (40) discovered that the clinician's objective scar rating differs significantly from the patient-rated scar severity in adult patients (>16 years) with heterogenous types of scars presenting to an outpatient department, and that the latter rather correlates with subjective psychological distress. Lowe (41) noticed that one of the most important predictors of parental satisfaction in pediatric laceration repair is “provider performance,” comprising communication, attitude, hygiene and confidence of the physician. Many studies naturally address the parental point of view, (41, 42) as self-reports are limited by age, but thus accept that self-reported health-related QoL potentially differs from proxy assessment (43). The CDLQI, which was used in the present population, is the most widely used instrument for measuring skin-related QoL in children (20). It provides evidence of the functional disability as well as its effects in terms of activities of daily living. In this study, the patient scale of the POSAS revealed low patient scores, indicating a good esthetic outcome in both groups, and CDLQI showed mild impairment of QoL at the early follow-up but no long-term effect. These results are consistent with the review of Martin-Herz (44), revealing improvement in health-related QoL seen at 6 months to 2 years after general traumatic injuries in pediatric individuals.

### Strengths and Limitations

The results are of particular significance because this is, to our knowledge, the largest single-center study in this field in children and the first to include objective scar assessment by surgeons blinded to the method of treatment. In contrast to other studies, this trial included a large number of validated assessment scales as well as the assessment of complications, costs, and time consumption of treatment, allowing for a comprehensive assessment of the two methods. Furthermore, the single-center study allowed all late follow-ups to be performed by the same person, thus enhancing the quality and comparability of the results as well as limiting observer variations. At the same time, the study has a number of limitations. The single-center observation may limit generalizability, as it is a unique setting in terms of experience and competency in the field. Another shortcoming is that most of the patients were Caucasian, impeding the assessment of esthetic outcomes and complication rates in other ethnicities. In particular, different ethnicities along with darker skin types might have impacted the results. Moreover, the number of patients included into one of the study groups was slightly below the number required according to the sample size calculation. Furthermore, the validated scar assessment tools applied in this study (POSAS and VSS) were not developed specifically for the use in linear scars, neither do they specifically evaluate the presence of suture marks and furthermore they were originally designed for in-patient scar assessment. From a methodological point of view, the most important limitation is the study's lack of randomization of the two treatment groups. This shortcoming has the potential to be a major source of bias: It is conceivable that treating physicians tend to apply skin adhesives in the less dehiscent, more superficial or for example, less macerated scar, which could potentially have a positive bias on scar outcomes, such as POSAS or VSS, and lead to a reduced rate in complications. However, performing randomization would have been ethically unacceptable in the present cohort, since suturing a wound in a young child often requires procedural sedation or even general anesthesia.

## Conclusion

The data shown in this study indicate that both treatment modalities lead to good long-term results and that complications are rare. At the same time, the application of adhesives seems to be less traumatizing, is safe, requires less anesthesia, and is more cost-effective than suturing. Furthermore, suturing or even the removal of the stitches following suturing can be a major challenge in children and lead to significant sequelae, as unfortunately experienced in one case in this cohort. Another advantage of skin adhesives—albeit not a specific question of this investigation—is that they can easily be applied in the doctor's office, whereas most of them do not have the means to perform suturing, often resulting in referrals to hospital. Therefore, in our opinion, skin adhesives should be strongly considered if not preferred over suturing in most situations. There are only few exceptions in which suturing remains favorable for repair of facial laceration: (1) when the risk of potential migration of tissue adhesives from the nearby wound into the eye is considerable (45) and (2) in large, frayed wounds, if the application of the tissue adhesive is assumed to be tricky.

## Data Availability Statement

The raw data supporting the conclusions of this article will be made available by the authors, without undue reservation.

## Ethics Statement

The studies involving human participants were reviewed and approved by Kantonale Ethikkommission Zürich, Stampfenbachstrasse 121, 8090 Zürich, Switzerland. Written informed consent to participate in this study was provided by the participants' legal guardian/next of kin.

## Author Contributions

SF designed the study and the data collection instruments, coordinated and supervised data collection, drafted the manuscript, and reviewed it for important intellectual content. CS and ML conceptualized and designed the study and critically reviewed the manuscript for important intellectual content. KN and GS conceptualized the study, reviewed, and revised the manuscript. SvS collected the data, carried out the initial analyses, and drafted the manuscript. JE conceptualized the study, carried out data interpretations and analysis, reviewed, and revised the manuscript. CM contributed to the analysis and interpretation of data for the work, revised and critically reviewed the manuscript. All authors approved the final manuscript as submitted and agree to be accountable for all aspects of the work.

## Conflict of Interest

The authors declare that the research was conducted in the absence of any commercial or financial relationships that could be construed as a potential conflict of interest.

## Abbreviations

CDLQI: Children's Dermatology Life Quality Index
CHF: Swiss franc
ED: emergency department
ICC: intraclass correlation coefficient
LET: lidocaine, epinephrine, tetracaine
POSAS: Patient and Observer Scar Assessment Scale
QoL: quality of life
VAS: Visual Analog Scale
VSS: Vancouver Scar Scale
WES: Wound Evaluation Score.
